# Effects of GSTM1/GSTT1 Gene Polymorphism and Fruit & Vegetable Consumption on Antioxidant Biomarkers and Cognitive Function in the Elderly: A Community Based Cross-Sectional Study

**DOI:** 10.1371/journal.pone.0113588

**Published:** 2014-11-24

**Authors:** Linhong Yuan, Weiwei Ma, Jinmeng Liu, Liping Meng, Jixia Liu, Shuang Li, Jing Han, Quanri Liu, Lingli Feng, Chao Wang, Rong Xiao

**Affiliations:** 1 School of Public Health, Capital Medical University, Beijing, P.R.China; 2 Beijing Key Laboratory of Environmental Toxicology, Capital Medical University, Beijing, P.R. China; 3 Institute of Nutrition and Food Safety, Chinese Center for Disease Control and Prevention, Beijing, P.R. China; 4 Nanyuan Community Health Service Center of Fengtai District, Beijing, P.R. China; Texas Tech University Health Science Centers, United States of America

## Abstract

**Background:**

It was reported that Glutathione S-transferase (GST) gene polymorphism and fruit and vegetable (FV) intake were associated with body antioxidant capacity. The oxidative/anti-oxidative imbalance played an important role in the pathogenesis of AD. However, the association of GST genotype, dietary FV consumption with body antioxidant biomarkers and cognitive function in the elderly is not clear.

**Objective:**

The aim of the present study was to determine the association of GST genotype, and dietary FV intake, with antioxidant biomarkers and cognitive function in the elderly.

**Methods:**

Food frequency questionnaire was used to collect data of dietary FV intakes in 504 community dwelling elderly aged from 55 to 75 years old. GSTM1 and GSTT1 genotypes were determined by using multiple-PCR method. Plasma and erythrocyte antioxidant biomarkers were measured. Cognitive function was measured by using Montreal Cognitive Assessment. Statistical analysis was applied for exploring the association of GST genotype and FV intake with antioxidant biomarkers level and cognitive function in the elderly.

**Results:**

Individual GSTM1 or GSTT1 gene deletion affects body antioxidant biomarkers levels, including erythrocyte GST activity, plasma total antioxidant capacity, and glutathione levels. GSTM1and/or GSTT1 gene deletion have no effects on cognitive function in the surveyed participants. The effect of GST genotype on antioxidant biomarkers are FV intake dependent. There is interaction of FV intake and GST genotype on cognitive function in the elderly.

**Conclusion:**

GST genotype or daily FV consumption impact body antioxidant biomarkers, but not cognitive function in the elderly. There were combined effects of GST genotype and FV consumption on cognitive function in the elderly population. Large scale perspective population study is required to explore the association of GST genetic polymorphism, FV consumption and antioxidant biomarkers and cognitive function in the elderly.

## Introduction

Alzheimer's Disease (AD) seriously affected the health of the elderly throughout the world. However, up to date, there are no effective treatments available for AD. Decline in cognitive function is one of the major symptoms of AD [1]. Individuals with a worse cognitive function, and/or a more rapid cognitive decline over time have a higher risk of development of AD [2]. As a result, to treat mild cognitive impairment and to prevent the subjects with cognitive function decline to further develop into AD was a promising strategy to control the prevalent of AD.

The benefits of high FV consumption are well known [3]. Increased intake of FV may provide defense against oxidative stress [4]. Additionally, intake of FV derived dietary antioxidants has been proved associating with decreased risk of variety of oxidative damage related chronic diseases, such as cancer, cardiovascular diseases, and AD [5], [6]. Although the association between FV intake and body antioxidant capacity appears well established. However, the underlying mechanisms of how to prevent oxidative damage related chronic diseases by high intakes of FV are not fully understood.

Glutathione S-transferase (GST) are family phase II enzymes involved in the detoxification of endogenous and exogenous electrophilic compounds, and the metabolism of compounds formed during oxidative stress [7]. Polymorphisms have been identified in the genes coding for GST family enzymes. The absence of enzyme function caused by gene deletion was proved contributing to the inter-individual differences in response to an increased susceptibility to oxidative stress related diseases [8]–[11]. Besides, individuals with GSTM1 and GSTT1 null genotypes were reported better protected from lung cancer than those with GSTM1/GSTT1 positive genotypes when exposing to cruciferous rich diets [12]. Therefore, it was suggested that subjects with an unfavorable polymorphism would be more susceptible to oxidative damage and possibly will benefit more from protection by dietary antioxidants [13].

Oxidative damage begins early in the progress of the AD [14]. Accumulation of products of free radical damage in the central nervous system and in the peripheral tissues of subjects with AD or mild cognitive impairment (MCI) was also proved [15], [16]. As a result, oxidative damage represents a potential therapeutic target for slowing the onset and progression of AD. Recently, the combined effects of genetic background with environmental factors, such as diet, on the risk of chronic diseases have been caused more and more extensive attention [17], [18]. Recent epidemiological studies also indicated that GSTM1 null and GSTT1 null genotypes were correlated with an increased susceptibility to diseases associated with oxidative stress, such as cancer, cardiovascular and respiratory diseases [19]–[21]. It is also reported that the frequency of GSTM1-null and GSTT1-null genotype was 35.7%–66% and 58%–66.5% respectively in Chinese population [22]. Our previous study also detected the combined effects of GST genotype and dietary FV consumption on antioxidant biomarkers in the health adults [10]. Therefore, in the present we mainly explore the impact of fruit and vegetable consumption, GSTM1/GSTT1 gene polymorphism on antioxidant biomarkers and cognitive function in the elderly.

Therefore, the aim of the current study was to verify the association of genetic background (GST genotype) and environmental factor (dietary FV intake) with antioxidant parameters and cognitive function in elderly population. The present study will contribute to the strategy making of genetic background-based dietary guideline for preventing MCI and AD in the elderly.

## Methods

### 1. Participants

**Figure 1:**
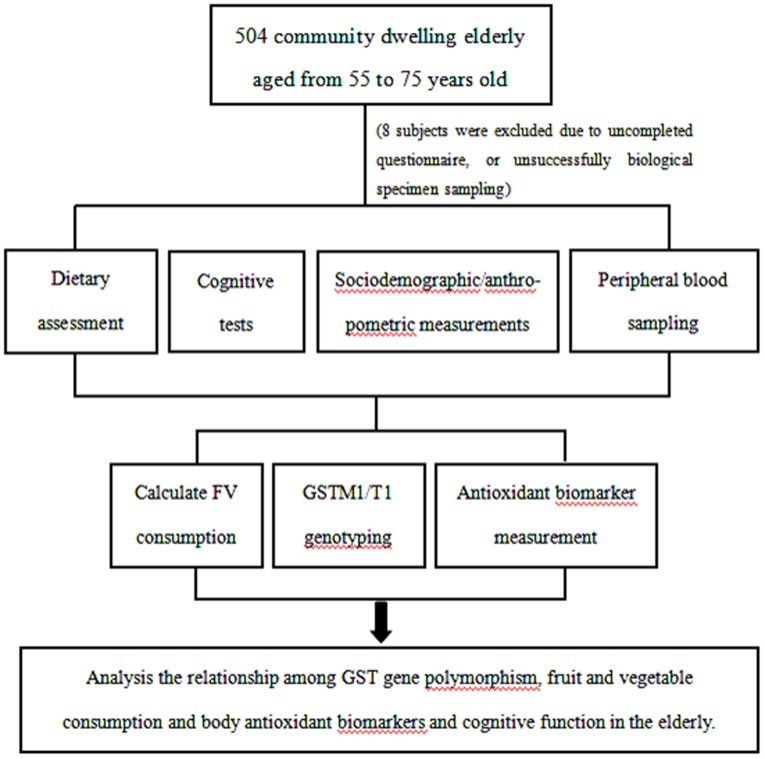
Graphic presentation of the overall design.

The study was a community based cross-sectional study, and the overall study design and sampling scheme are shown in **Figure 1**. The study protocol was approved by the Human Ethics Committee of the Capital Medical University (No. 2012SY23). A total of 504 community dwellers aged 55–75 was selected. Criteria for exclusion of the subjects were conditions known to affect biological variables of oxidative stress or cognitive function (e.g. inflammatory diseases, recent history of heart or respiratory failure, chronic liver or renal failure, malignant tumors, a recent history of alcohol abuse, history of cerebral apoplexy, history of cerebral infarction). All participants gave written informed consent.

### 2. Dietary assessment

Participants were visited at community health service center by specifically trained dieticians. A validated self-administered semi-quantitative food frequency questionnaire (FFQ) was used to assess the habitual consumption of 11 food items (totally including 41 questions). This questionnaire was adapted from the questionnaire used for the Dietary Investigation of Chinese Residents, which was organized by the Chinese Nutrition Society (CNS) in 2010. FV intake survey collected the information including the consumption frequencies of FV, quantity of FV consumed per day, and variety of FV consumed per week.

### 3. Cognitive tests

Cognitive tests were carried out by using Montreal Cognitive Assessment (MoCA) by trained investigators and took about 15 min to complete. The tests have also been widespread used in other large-scale studies on cognitive function in the elderly.

### 4. Sociodemographic variables and anthropometric measurements

Anthropometric measures (height and weight) were made by the nurse in the community medical service center, and BMI was calculated as weight (kg)/height(m)^2^. Information on demographic characteristics (for example, age, education and marital status), lifestyle factors (for example, alcohol drinking, smoking and physical activity), medical history of chronic diseases and dietary supplements was collected using a self-administered questionnaire. Educational level was assessed as the highest level reached and classified into four categories (illiterate, primary school, junior high school, and >high school). Smoking status was defined as being a non-smoker or current smoker. Physical activity level was classified into three categories: inactive (no physical activity), moderately active (1–3 times per week), and active (everyday). Questions on alcohol consumption were about intakes of beer, wine, and spirits during the week.

### 5. Other covariates

Sex and race were obtained at the time of the census and verified at interview. Age was computed from self-reported birth date. Participation in cognitive activities was reading newspapers, books, or magazines, listening to the radio, computer using and watching TV.

### 6. DNA isolation and genotyping

Peripheral blood samples (6 ml venipuncture) were collected in heparinized vacutainers and stored at −80°C. DNA was extracted from frozen peripheral heparinized blood using the Wizare genomic DNA purification kit (Promega, Wisconsin, USA). GSTT1 and GSTM1 genotypes were determined by multiple polymerase chain reaction (PCR) method using primers and reaction profiles as described by Zhong [23], [24] respectively. The primers for the analyzed systems are: GSTM1 primers (Accession: NM_000561.3): 5′-GAA CTC CCT GAA AAG CTA AAG C-3′; 5′-GTT GGG CTC AAA TAT ACG GTG G-3′; GSTT1 (Accession: NM_000853.2): 5′-TTC CTT ACT GGT CCT CAC ATC TC-3′; 5′-TCA CCG GAT CAT GGC CAG CA-3′; β-globin (Accession: M11428.1): 5′-GAA GAG CCA AGG ACA GGT AC-3′; 5′-CAA CTT CAT CCA CGT TCA CC-3′. GSTM1 and GSTT1 null genotypes were determined by identifying the negative band for each size (with the simultaneous presence of the positive control), while positive bands meant the sample was homozygous or heterozygous for the indicated alleles. The genotype with homozygous deletion of the GST genes is called “*GST−*”, whereas the genotype having at least one copy of the gene is called “*GST+*”. To ensure genotyping reliability, approximately 10% of the samples were randomly selected and genotyped independently using the same protocol. In all cases, the outcome was concordant.

### 7. Plasma antioxidant biomarker measurement

For the antioxidant biomarker analysis, blood samples were centrifuged at 480 g for 20 min. The plasma was then removed (top layer) and transferred to storage tubes and frozen at −80°C.

Plasma total antioxidant capacity (T-AOC) were measured using commercial assay kits (Nanjing Jiancheng Institute, China) according to the manufacturer's instruction. Briefly, Fe^3+^ can be reduced to Fe^2+^, the latter with the Philippine-solid substances to form complexes by colorimetric assay can measure T-AOC activity. Two independent measurements were performed for each sample. T-AOC was expressed as U/ml.

Plasma glutathione (GSH) content was determined using a commercial kit (Nanjing Jiancheng Institute, China) according to the manufacturer's instruction. Briefly, 0.5 mL of plasma was added to 1 mL of 0.4 M TRIS buffer, pH 8.9. Finally, 25 µL of DTNB (5, 5′-dithiobis-2-nitro benzoic acid) was added to react with GSH. A standard curve was performed in order to determine GSH concentration in samples. Two independent measurements were performed for each sample. The absorbance was read at 412 nm, and results were expressed as µmol/L.

### 8. Erythrocyte antioxidant enzyme activity measurement

Erythrocyte catalase (CAT) activity was determined by measuring the intensity of a yellow complex formed by molybdate and hydrogenperoxide (H_2_O_2_) at 405 nm after adding ammonium molybdate to terminate H_2_O_2_ degradation, which is catalyzed by CAT. One unit of CAT activity is expressed as the degradation of 1.0 µmol/L H_2_O_2_ per second per 1.0 mL reaction solution. Two independent measurements were performed for each sample. CAT activity was normalized per microgram of hemoglobin.

Superoxide dismutase (SOD) activity in erythrocyte was measured through the inhibition of nitroblue tetrazolium reduction by the superoxide radicals generated by the xanthine/xanthine oxidase system. One unit of SOD activity is defined as the enzyme amount causing 50% inhibition in 1.0 mL reaction solution. Two independent measurements were performed for each sample. SOD activity was normalized per microgram of hemoglobin.

Erythrocyte glutathione peroxidase (GSH-Px) activity was determined by measuring the reduction of GSH per minute on the base of its catalysis. The final results were expressed as a decrease of 1.0 µmol/L GSH per 5 minutes for 0.1 mL hemolysate at 37°C after the nonenzymatic reaction is subtracted. Two independent measurements were performed for each sample. GSH-Px activity was normalized per microgram of hemoglobin.

Erythrocyte total GST enzyme activity was measured spectrophotometrically using 1-chloro-2,4-dinitrobenzene (CDNB, Sigma Aldrich, USA) as a substrate according to the method of Habig et al. [25]. One unit of GST activity represents the formation of 1 µmol of CDNB-GSH per minute of incubation. Two independent measurements were performed for each sample. GST activity was normalized per microgram of hemoglobin.

Erythrocyte glutathion reductase (GR) activity was determined at 340 nm using a commercial kit (Nanjing Jiancheng Institute, China) according to the manufacturer's instruction. One activity unit was defined as 1 µmol NADP (NADPH) formed per minute per 1.0 mL hemolysate. Two independent measurements were performed for each sample. GR activity was normalized per gram of hemoglobin.

### 9. Statistical analyses

All statistical analyses were performed with SPSS 19.0 Statistical package. Participants were classified according to categories of GST genotypes and daily FV intake levels. Demographic characteristics were compared between categories of the GST genotypes. A general linear model was used to compare the antioxidant parameters between GST genotypes or daily FV consumption. This statistical method has been used widely to analyze effects of gene polymorphism and environmental exposure on serum activity [26], [27]. Some potential confounding factors including sex, age, BMI, smoking and antioxidant supplementation were adjusted when comparing the parameters between different GST genotypes or different daily FV consumption. The potential confounding factors including the sex, age, BMI, family history of AD, education levels, physical activity, and participation in cognitive activities were adjusted when comparing the difference between GST genotypes and daily FV consumption. A level of significance was set at a *P* value of less 0.05.

According to the data of Dietary Survey in China carried out by the Chinese Nutrition Society (CNS) in 2010 [28], we categorize the subjects into low, middle, and high FV consumption groups. CNS reported that the average fruit intake of Chinese adults is 71.8 g/d, and the average vegetables intake is 264.3 g. Therefore, in the current study, we classify daily FV consumption into 3 categories: low, <50 g per day; middle, 50–100 g per day; high, >100 g per day. For vegetables intake, we categorize the options into 3 levels: low, <150 g per day; middle, 150–350 g per day; high, >350 g per day. Besides, according to the Dietary Guideline of Chinese recommended by CNS, in which, 150–200 g fruit and 400–500 g vegetables were recommended for Chinese adults. Therefore, in the current study, we categorized daily FV consumption less than 200 g per day as the low FV intake group; and FV consumption more than 500 g per day as the high FV intake group.

## Results

### 1. Demographic characters of the participants

Totally, 504 subjects participated in the dietary survey, and 8 subjects were excluded due to uncompleted questionnaire, or unsuccessfully biological specimen sampling. GSTM1/GSTT1 variants, demographic and lifestyle characteristics of the study population were shown in **Table 1**. GSTM1/GSTT1 genotypes of 496 participants were distributed as follows: 24.80% GSTM1+/GSTT1+ (n = 123), 21.57% GSTM1+/GSTT1− (n = 107), 29.03% GSTM1−/GSTT1+ (n = 144), and 24.60% GSTM1−/GSTT1− (n = 122). There was no statistical significance of age and BMI among the subjects with different GST genotypes (*P*>0.05).

**Table 1:** Unadjusted demographic characteristics of the participants.

All (*n = 496*)		GST genotype			
		GSTM1+/GSTT1+ (*n = 123*)	GSTM1+/GSTT1− (*n = 107*)	GSTM1−/GSTT1+ (*n = 144*)	GSTM1−/GSTT1− (*n = 122*)
***Demographics***					
Age, y (mean ± SD)	63.22±5.35	63.24±5.54	63.58±5.87	63.49±5.16	62.58±4.90
Sex, male/female	177/319	53/70	37/70	42/102	45/77
Nationality, n (%)					
Han	468	115	101	135	117
Hui	17	7	4	5	1
MongoljEn	1	0	0	0	1
Manchu	10	1	2	4	3
BMI (kg/m^2^)	26.85±3.27	25.23±3.53	25.33±3.15	25.29±3.13	25.27±3.31
Education					
Illiterate	14	4	4	4	2
Primary school	43	13	8	20	2
Junior high school	233	58	57	59	59
>High school	206	48	38	61	59
***Life style factors***					
Physical activity					
Inactive	66	20	14	14	18
Moderately active	55	15	17	12	11
Active	375	94	82	99	100
Current smoker, n (%)	73 (14.72)	14 (2.82)	17 (3.43)	19 (3.83)	23(4.64)
Current alcohol, n (%)	117 (23.59)	33(6.65)	28(5.65)	26(5.24)	30(6.05)

The data of age and BMI were expressed as mean ± SE. BMI: body mass index. One-way ANOVA analysis was used to analyze the means of age and BMI.

### 2. Antioxidant parameters and cognitive function according to GST genotype

As shown in **Table 2**, deletion of GSTM1 gene has no effect on erythrocyte GSH-Px, GST, GR, SOD and CAT enzyme activities and plasma T-AOC level (*P*>0.05). When comparing with GSTM1 gene positive subjects, GSTM1 gene deletion significantly decrease the content of GSH in plasma, and statistical significance was detected (*P*<0.01). The deletion of GSTT1 mainly influenced erythrocyte GST enzyme activity and plasma T-AOC levels. The elderly subjects with GSTT1− genotype have higher erythrocyte GST enzyme activity than the subjects with GSTT1+ genotypes (*P*<0.05). Additionally, the subjects with GSTT1− genotype have higher plasma T-AOC (*P*<0.05) than the subjects with GSTT1+ genotype (*P*<0.05). GSTM1 or GSTT1 gene deletion has no effect on cognitive function in the elderly (*P*>0.05).

**Table 2:** Antioxidant parameters and cognitive function according to individual GST genotype.

Parameters & cognitive function	GSTM1+ (*n = 196*)	GSTM1− (*n = 242*)	*P* value	GSTT1+ (*n = 224*)	GSTT1− (*n = 210*)	*P* value
**Erythrocyte**						
GSH-Px (U/gHb)	26.34±7.39	25.63±8.06	0.348	25.29±6.91	26.64±8.54	0.071
GST (U/gHb)	0.33±0.129	0.34±0.17	0.251	0.32±0.16	0.35±0.15	0.034
GR (U/gHb)	0.57±0.18	0.55±0.19	0.296	0.55±0.19	0.56±0.19	0.319
CAT (U/gHb)	2.36±0.43	2.36±0.36	0.950	2.36±0.42	2.35±0.36	0.716
SOD (U/gHb)	31.84±4.77	31.32±4.53	0.239	31.50±4.48	31.61±4.82	0.694
**Plasma**						
T-AOC (activity U/L)	11.55±4.72	11.51±3.79	0.977	11.09±3.67	12.00±4.71	0.019
GSH (µmol/L)	58.72±8.78	48.78±9.48	0.007	50.90±11.96	54.07±10.50	0.524
**MoCA score**	26.38±3.09	26.49±2.86	0.969	26.44±2.97	26.43±2.96	0.694

General linear model used. Data are presented as the mean ± SE. For antioxidant biomarker data analysis, factors including age, sex, BMI, smoking and antioxidant supplement were adjusted. For cognitive function (MoCA score) data analysis, factors including age, sex, BMI, education, family history of AD, physical activity and participation in cognitive activity were adjusted. GSH-Px, glutathione peroxidase; GST, glutathione S-transferase; GR, glutathion reductase; CAT, catalase; SOD, superoxide dismutase; GSH, glutathione; T-AOC, total antioxidant capacity; MoCA, Montreal Cognitive Assessment. *P* value<0.05 was considered as significant.

### 3. Antioxidant parameters and cognitive function according to FV consumption

Daily FV intake has no effect on the detected erythrocyte antioxidant enzyme activities (*P*>0.05) and cognitive function (*P*>0.05) in the elderly. FV intake also did not affect plasma GSH content in the elderly (*P*>0.05). While, individuals in FV intake less than 200 g category have the lowest mean plasma T-AOC level comparing with other categories (*P*<0.05) (**Table 3**).

**Table 3:** Antioxidant parameters and cognitive function according to FV intake.

Parameters	FV intake (g/d)			*P* value
	<200 (n = 37)	200–500 (n = 195)	>500 (n = 206)	
**Erythrocyte**				
GSH-Px (U/gHb)	27.49±9.21	25.65±7.49	25.94±7.75	0.496
GST (U/gHb)	0.30±0.17	0.35±0.15	0.33±0.15	0.153
GR (U/gHb)	0.54±0.20	0.58±0.19	0.54±0.18	0.126
CAT (U/gHb)	2.41±0.35	2.35±0.37	2.35±0.42	0.700
SOD (U/gHb)	31.89±4.39	31.28±5.15	31.75±4.17	0.393
**Plasma**				
T-AOC (activity U/L)	10.02±3.78	11.49±4.70	11.87±3.71	0.043
GSH (µmol/L)	55.85±13.09	54.88±11.70	49.54±9.39	0.303
**MoCA score**	26.85±3.06	26.18±3.23	26.65±2.65	0.207

General linear model used. Data are presented as the mean ± SE. For antioxidant biomarker data analysis, factors including age, sex, BMI, smoking and antioxidant supplement were adjusted. For cognitive function (MoCA score) data analysis, factors including age, sex, BMI, education, family history of AD, physical activity and participation in cognitive activity were adjusted. FV, fruit and vegetable; GSH-Px, glutathione peroxidase; GST, glutathione S-transferase; GR, glutathion reductase; CAT, catalase; SOD, superoxide dismutase; GSH, glutathione; T-AOC, total antioxidant capacity; MoCA, Montreal Cognitive Assessment. *P* value<0.05 was considered as significant.

### 4. Antioxidant parameters and cognitive function according to FV consumption and individual GST genotype

As shown in **Table 4**, no combined effects of FV intake and GSTM1 genotype on erythrocyte GSH-Px, GR, SOD, CAT enzyme activities and plasma T-AOC, GSH levels (*P*>0.05). The effect of GSTM1 genotype on erythrocyte GST enzyme activity was FV intake dependent. The GSTM1+ elderly subjects in the FV intake less than 200 g category have the lower erythrocyte GST enzyme activity comparing with that of GSTM1− elderly subjects (*P*<0.05); while, when the FV consumption increased to more than 500 g per day, there was no significant difference between the categories (*P*>0.05). No combined effect of FV intake and GSTM1 genotype on cognitive function in the elderly (*P*>0.05). Daily FV consumption and GSTT1− genotypes have no effects on erythrocyte antioxidant enzyme activities (*P*>0.05) and plasma GSH levels (*P*>0.05). The plasma T-AOC level exhibits FV consumption and GSTT1 genotype dependent trend, and the GSTT1+ subjects in FV consumption less than 200 g category have the lower plasma T-AOC level than that of the GSTT1− subjects (*P*<0.05). Moreover, there is interaction between daily FV intake and GSTT1 genotypes on cognitive function in the elderly (*P _FV and GSTT1 genotype_*<0.05).

**Table 4:** Antioxidant parameters and cognitive function according to FV consumption and individual GST genotype.

Biomarkers		FV<200 (n = 37)		FV>500 (n = 206)		FV<200 (n = 37)		FV>500 (n = 206)	
		GSTM1+ (n = 16)	GSTM1− (n = 21)	GSTM1+ (n = 88)	GSTM1− (n = 118)	GSTT1+ (n = 18)	GSTT1− (n = 19)	GSTT1+ (n = 118)	GSTT1− (n = 88)
Erythrocyte	GSH-Px (U/gHb)	29.03±7.79	26.32±6.19	26.17±7.37	25.77±8.05	27.30±6.53	27.68±8.36	24.67±6.16	27.65±9.24
	GST (U/gHb)^a^	0.23±0.11	0.36±0.18	0.33±0.12	0.33±0.18	0.29±0.16	0.31±0.17	0.32±0.15	0.35±0.15
	GR (U/gHb)	0.53±0.19	0.55±0.21	0.53±0.17	0.55±0.19	0.49±0.15	0.59±0.23	0.53±0.18	0.55±0.18
	CAT (U/gHb)	2.36±0.46	2.44±0.25	2.34±0.44	2.36±0.41	2.51±0.37	2.31±0.31	2.36±0.43	2.35±0.41
	SOD (U/gHb)	30.36±2.88	33.05±5.02	31.99±3.40	31.57±4.67	31.90±3.27	31.88±5.33	31.32±3.60	32.33±4.79
Plasma	T-AOC (activity U/L)^b^	10.74±4.02	9.43±3.33	11.54±3.93	12.11±3.60	9.87±3.46	10.11±3.91	11.35±3.80	12.56±3.58
	GSH (µmol/L)	63.60±14.84	49.94±8.21	56.12±11.33	44.58±7.61	48.13±9.16	63.16±13.46	49.74±8.16	49.25±9.24
MoCA score^c^		27.63±2.50	26.14±3.31	26.81±2.54	26.50±2.72	27.47±2.65	26.05±3.33	26.31±3.10	27.07±1.80

General linear model used. Data are presented as the mean ± SE. For antioxidant biomarker data analysis, factors including age, sex, BMI, smoking and antioxidant supplement were adjusted. For cognitive function (MoCA score) data analysis, factors including age, sex, BMI, education, family history of AD, physical activity and participation in cognitive activity were adjusted. FV, fruit and vegetable; GSH-Px, glutathione peroxidase; GST, glutathione S-transferase; GR, glutathion reductase; CAT, catalase; SOD, superoxide dismutase; GSH, glutathione; T-AOC, total antioxidant capacity; MoCA, Montreal Cognitive Assessment. *P* value<0.05 was considered as significant.

a : GSTM1 genotype affect the erythrocyte GST enzyme activity, *P_GSTM1 genotype_* = 0.012, <0.05.

b : FV consumption affect the plasma T-AOC in the subjects with different GSTT1 genotypes, *P_FV_* = 0.04, <0.05.

c : Interaction of FV and GSTT1 genotype on cognitive function was detected, *P_FV and GSTT1 genotype interaction_* = 0.033, <0.05.

### 5. Antioxidant parameters and cognitive function according to combined GST genotype

Regarding to the combined effect of GSTM1 and GSTT1 genotype on body antioxidant parameters and cognitive function, no combined effects of GSTM1/GSTT1 on body antioxidant parameters in the elderly, including the erythrocyte antioxidant enzyme activities (*P*>0.05) and plasm T-AOC and GSH levels (*P*>0.05). In addition, we did not detect the difference of the cognitive function among the subjects with different GSTM1/GSTT1 genotypes (*P*>0.05) (**Table 5**).

**Table 5:** Antioxidant parameters and cognitive function according to combined GST genotype.

Parameters & cognitive function	Genotype				*P* value
	GSTM1+/GSTT1+ (n = 97)	GSTM1+/GSTT1− (n = 99)	GSTM1−/GSTT1+ (n = 128)	GSTM1−/GSTT1− (n = 114)	
**Erythrocyte**					
GSH-Px (U/gHb)	25.35±6.75	27.30±7.88	25.24±7.06	26.06±9.06	0.188
GST (U/gHb)	0.31±0.12	0.34±0.13	0.33±0.18	0.36±0.16	0.098
GR (U/gHb)	0.54±0.17	0.59±0.18	0.55±0.19	0.54±0.19	0.104
CAT (U/gHb)	2.38±0.48	2.33±0.38	2.35±0.38	2.37±0.34	0.795
SOD (U/gHb)	31.82±4.74	31.87±4.83	31.26±4.27	31.38±4.83	0.628
**Plasma**					
T-AOC (activity U/L)	11.20±4.15	11.89±5.22	11.00±3.28	12.10±4.24	0.144
GSH (µmol/L)	58.80±15.34	58.64±12.23	44.87±8.28	50.15±10.78	0.057
**MoCA score**	26.79±2.64	26.11±3.47	26.32±2.92	26.77±2.45	0.351

General linear model used. Data are presented as the mean ± SE. For antioxidant biomarker data analysis, factors including age, sex, BMI, smoking and antioxidant supplement were adjusted. For cognitive function (MoCA score) data analysis, factors including age, sex, BMI, education, family history of AD, physical activity and participation in cognitive activity were adjusted. GSH-Px, glutathione peroxidase; GST, glutathione S-transferase; GR, glutathion reductase; CAT, catalase; SOD, superoxide dismutase; GSH, glutathione; T-AOC, total antioxidant capacity; MoCA, Montreal Cognitive Assessment. *P* value<0.05 was considered as significant.

### 6. Antioxidant parameters and cognitive function according to FV consumption and combined GST genotype

The data of **Table 6** show that, subjects, in the FV intake less than 200 g category with GSTM1+/GSTT1+ or GSTM1+/GSTT1−, have the lower erythrocyte GST enzyme activity than that of the GSTM1−/GSTT1+ and GSTM1−/GSTT1− elderly subjects (*P _genotype_* = 0.036, <0.05). When the daily FV consumption increased to more than 500 g per day, the effect of GSTM1/GSTT1 genotype on GST enzyme activity was not significant (*P*>0.05). There was no combined effects of FV consumption and GSTM1/GSTT1 genotype on GSH-Px, GR, SOD, CAT enzyme activities and plasma GSH and T-AOC levels (*P*>0.05). GSTM1/GSTT1 genotype or daily FV consumption have no effect on cognitive function, however, we detected the interaction of FV consumption and GST genotype on cognitive function in the elderly (*P _FV and genotype_*<0.01). When daily FV consumption less than 200 g, the GSTM1−/GSTT1− subjects have the lowest MoCA score; however, when daily FV consumption increased to more than 500 g per day, there is no significant difference of cognitive function among the subjects with different GSTM1/GSTT1 genotypes (*P*>0.05).

**Table 6:** Antioxidant parameters and cognitive function according to FV consumption and combined GST genotype.

Biomarkers	FV (g/d)							
	<200 (n = 37)				>500 (n = 206)			
	GSTM1+/GSTT1+ (n = 8)	GSTM1+/GSTT1− (n = 8)	GSTM1−/GSTT1+ (n = 10)	GSTM1−/GSTT1− (n = 11)	GSTM1+/GSTT1+ (n = 56)	GSTM1+/GSTT1− (n = 32)	GSTM1−/GSTT1+ (n = 30)	GSTM1−/GSTT1− (n = 28)
**Erythrocyte**								
GSH-Px (U/gHb)	26.73±5.28	31.33±9.48	27.75±7.64	25.02±6.29	25.23±6.52	27.81±8.52	24.17±5.82	27.55±8.71
GST (U/gHb)^a^	0.23±0.10	0.22±0.13	0.35±0.19	0.37±0.17	0.32±0.11	0.34±0.13	0.31±0.18	0.36±0.16
GR (U/gHb)	0.46±0.13	0.60±0.23	0.51±0.17	0.58±0.24	0.52±0.18	0.54±0.16	0.53±0.19	0.56±0.19
CAT (U/gHb)	2.50±0.47	2.22±0.43	2.52±0.30	2.37±0.19	2.36±0.44	2.31±0.44	2.36±0.43	2.37±0.40
SOD (U/gHb)	31.50±2.26	29.23±3.11	32.23±4.00	33.81±5.88	31.44±3.24	32.95±3.51	31.20±3.92	31.97±5.39
**Plasma**								
T-AOC (activity U/L)	10.33±3.30	11.15±4.83	9.51±3.72	9.36±3.12	11.20±4.15	11.89±5.22	11.00±3.28	12.10±4.24
GSH (µmol/L)	44.98±7.64	62.22±12.34	50.64±11.50	49.31±9.88	60.41±12.27	48.63±8.88	40.11±5.58	49.62±9.42
MoCA Score^b^	28.00±1.93	28.00±2.89	26.90±3.21	24.80±3.29	26.77±2.84	26.94±2.06	25.87±3.29	29.19±1.67

General linear model used. Data are presented as the mean ± SE. For antioxidant biomarker data analysis, factors including age, sex, BMI, smoking and antioxidant supplement were adjusted. For cognitive function (MoCA score) data analysis, factors including age, sex, BMI, education, family history of AD, physical activity and participation in cognitive activity were adjusted. FV, fruit and vegetable; GSH-Px, glutathione peroxidase; GST, glutathione S-transferase; GR, glutathion reductase; CAT, catalase; SOD, superoxide dismutase; GSH, glutathione; T-AOC, total antioxidant capacity; MoCA, Montreal Cognitive Assessment. *P* value<0.05 was considered as significant.

a : *P _genotype_* = 0.036, <0.05. *P_FV and genotype_* = 0.318, >0.05;

b : *P_FV_* = 0.384, *P _genotype_* = 0.413; *P_FV and genotype_* = 0.004, <0.01.

## Discussion

In the current study, we focused on the relationship between dietary FV consumption, GST genetic polymorphism and antioxidant parameters in the elderly. In particular, we investigated the combined effects of FV consumption and GST genotype on cognitive function in the elderly. Our study highlighted a significant association between GSTM1 or GSTT1 gene deletion and erythrocyte GST enzyme activity and plasma GSH levels. Daily FV consumption impacted the plasma T-AOC in elderly subjects. There was no association among FV consumption, GST genotype and MoCA total score in the elderly. The interaction of FV consumption and GSTM1 and/or GSTT1 genotype on erythrocyte GST activity, plasma T-AOC and MoCA total score in the elderly was present.

Our data indicated that the deletion of GSTM1 or GSTT1 gene has no effects on erythrocyte GSH-PX, GR, CAT and SOD enzyme activities. These results were consistent with Sahar S. Bessa et al's study in Egyptian patients with essential hypertension [29], and Lakhdar R. et al's study in the patients with chronic obstructive pulmonary disease [30]. Not as we observed in healthy young adults (in them the GST activity performing as GSTM1 genotype dependent) [31], GSTT1 deletion significantly increased the GST activity (*P* = 0.034, <0.05) in the elderly. Additionally, we did not observed the combined effects of GSTM1 and GSTT genes on GST activity as well as we found in the healthy young adults.

The GST genotype did not effect plasma T-AOC levels in the elderly. The data are in line with our [31] and other's reported. Martin [32] and Dušinská [33] reported that there was no difference among the individuals with different GSTM1/GSTT1 genotypes in plasma T-AOC and other anti-oxidative biomarkers. However, Tang [34] and Mustafa's [35] found that individuals with GSTM1 and/or GSTT1 gene deletion had lower T-AOC level and higher oxidative stress than subjects with other GST genotypes. Lower plasma GSH level was found in GSTM1− elderly subjects (Table 2). Jian-Jin Tang et al's study of coronary artery disease (CAD) patients indicated that GSTM1 genotype have no effect on plasma GSH levels, and the deletion of GSTTT1 increased the plasma GSH levels in the CAD patients [36]. We still can not explain the accurate reasons for these different results. Such observations might be explainable by the difference of sample number included in each study; or the different methods used for biomarker measurement; or the population selected in the studies: the healthy adults (aged from 20 to 23 years old) *vs.* the elderly (aged from 55 to 75 years old), or the healthy elderly *vs.* the elderly with chronic diseases (such as hypertension, chronic obstructive pulmonary disease, and CAD).

The polymorphism GST family member genes have been previously reported to influence the age at onset of AD [37]. In the present study, we did not detected the influence of GSTM1 or/and GSTT1 deletion on cognitive function in the elderly (Table 2 and 5). These results are consistent with Gianfranco Spalletta's study, in which, the authors did not find any association between cognitive or functional outcome and the GSTM1 and GSTT1 deleted genotypes [38]. Ki-Do Eum and co-workers also did not observe the association of GSTM1 deletion polymorphism and the lead related cognitive function decline [39]. On the other hand, Pinhel's [40] study indicated that the nullity of GSTT1 gene proved to be additional risk factors for late-onset AD, and the presence of GSTT1 may indicate protection against late-onset AD. While, no association was identified between the late-onset AD with the GSTM1 polymorphism. The suggested mechanisms contributing to these contradictory results might be the disequilibrium expression or different localizations of GST family genes [41], [42]. Another possible explanation for these observations might be the modifying effects of environmental factors on the net contribution of genetic factors on health.

The effects of dietary FV intake on body antioxidant capacity have been extensively reported [43]. Since serum antioxidants, such as beta-carotene, vitamin C and polyphenols concentrations were positively correlated with the consumption of FV [44], [45], therefore we speculate that the variety of plasma T-AOC of the subjects in different FV consumption categories may attribute to the different content of plasma antioxidants content caused by different FV consumption.

Recently studies found that subjects who consume higher levels of FV scored higher on cognitive and neuropsychological evaluations [46], [47]. While, in the present study, we did not detected the relationship between daily FV intake on cognitive function in the elderly. In two studies, higher vegetable intake, but not fruit intake, was associated with less cognitive decline [48], [49]. Besides, the consumption of FV has been associated with decreased risk of dementia and AD [50]. Although effect of FV consumption on cognitive function was not detected in the present study, however, we found the the frequency of FV consumption and the variety of fruit consumed influence the cognitive function (data not shown). Ye X et al. also found that greater variety, but not total quantity, of FV intake was associated with a higher mini-mental state examination (MMSE) score in the elderly after multivariate adjustment [51]. This association remained significant after further adjusting for total quantity of fruit and vegetable intake. Further study is required to explore the relationship between dietary FV intake and cognitive function in the elderly.

We also fount that the influence of GSTM1 gene deletion on erythrocyte GST activity was FV intake dependent. And the results indicated that the deletion of GSTM1 or GSTT1 gene seems to be preventive factor for the individuals to the adverse environment (low FV intake). Under stress condition (low FV intake), the GSTM1+ subjects have lower erythrocyte GST enzyme activity than GSTM1− subjects; and the GSTT1+ subjects have lower plasma T-AOC levels than that of GSTT1− subjects. While, when the daily FV consumption increased to more than 500 g per day, the effects of GST gene deletion on antioxidant parameters were not remarkable. The lower GST enzyme activity was also detected in the GSTM1+/GSTT1+ under condition of low FV consumption. After increased the FV consumption, the difference among the group became not significant. The deletion of GSTM1 or/and GSTT1 was reported causing deficiency of GSTM1 and GSTT1 protein expression, therefore, negative modifying the GST activity. However, in the present study, the data seemed to hint that the deletion of these two genes was favorable to keep GST activity in erythrocyte. It is suggested that the lack of GSTT1 and GSTM1 genes might influence the expression of other genes. Earlier studies have shown that GSTM1 genotype status may influence other enzyme activities including GSTA [52]. A recent study showed that GSTT1 activities can be up-regulated by GSTM1 null genotypes upon exposure to pesticides [53]. These findings indicated a self-prevention mechanism of the body: under adverse condition, other GST family genes might be activated to complement the absence of GSTM1 or/and GSTT1 genes.

The interaction of GST genotype and FV consumption on the MoCA total score in the elderly was observed. Our results also indicated that FV intake may modify the effect of GST genotype on cognitive function in the elderly. These results further indicated the interaction of genetic background (GST genotype) with environmental factor (dietary FV intake). To some extent, these findings might explain the negative results (Table 2 and Table 3) during analyzing the individual effect of FV intake or GST genotype on MoCA total score in the elderly.

There are some limitations in the present study, for example, the small sample size, fail in collecting 24-hour dietary recall data to calculate the nutrients intake, and fail in detecting the content of antioxidants in the plasma etc. Despite of these limitations, the present study confirm the role of FV consumption and GST genotype in affecting antioxidant parameters, and highlighting the interaction of FV consumption and GST genotype on cognitive function in the elderly.
